# Trend on dental caries status and its risk indicators in children aged 12 years in China: a multilevel analysis based on the repeated national cross-sectional surveys in 2005 and 2015

**DOI:** 10.1186/s12889-021-12262-x

**Published:** 2021-12-15

**Authors:** Fei Li, Si-Cheng Wu, Zhi-Yuan Zhang, Edward Chin Man Lo, Wen-Jia Gu, Dan-Ying Tao, Xing Wang, Bao-Jun Tai, De-Yu Hu, Huan-Cai Lin, Bo Wang, Yan Si, Chun-Xiao Wang, Shu-Guo Zheng, Xue-Nan Liu, Wen-Sheng Rong, Wei-Jian Wang, Xi-Ping Feng, Hai-Xia Lu

**Affiliations:** 1 Department of Preventive Dentistry, Shanghai Ninth People’s Hospital, Shanghai Jiao Tong University School of Medicine, College of Stomatology, National Center for Stomatology, National Clinical Research Center for Oral Diseases, Shanghai Key Laboratory of Stomatology, Shanghai Jiao Tong University, Shanghai, China; 2 Biostatistics Office of Clinical Research Unit, Shanghai Ninth People’s Hospital, School of Medicine, Shanghai JiaoTong University, Shanghai, China; 3 Department of Oral and Maxillofacial-Head Neck Oncology, Shanghai Ninth People’s Hospital, Shanghai Jiao Tong University School of Medicine, College of Stomatology, National Center for Stomatology, National Clinical Research Center for Oral Diseases, Shanghai Key Laboratory of Stomatology, Shanghai JiaoTong University, Shanghai, China; 4 Dental Public Health, Faculty of Dentistry, University of Hong Kong, Pok Fu Lam, Hong Kong; 5 Chinese Stomatological Association, Beijing, China; 6 School & Hospital of Stomatology, Wuhan University, Wuhan, China; 7 West China School of Stomatology, Sichuan University, Chengdu, China; 8 Guanghua School of Stomatology, Hospital of Stomatology, Sun Yat-sen University, Guangzhou, China; 9 Chinese Center for Disease Control and Prevention, Beijing, China; 10 Department of Preventive Dentistry, National Engineering Laboratory for Digital and Material Technology of Stomatology, Beijing Key Laboratory of Digital Stomatology, Peking University School and Hospital of Stomatology, Beijing, China

**Keywords:** dental caries, cross-sectional studies, dental public health, epidemiology, multilevel analysis

## Abstract

**Background:**

This study aimed to explore the trend and risk indicators for dental caries of children aged 12 years in China based on national oral health survey data in 2005 and 2015.

**Methods:**

Research data were from the two latest national oral health surveys conducted in mainland China, including 30 and 31 provinces, autonomous regions, and municipalities in 2005 and 2015, respectively. Children aged 12 years were clinically examined for dental caries and dental fluorosis according to the World Health Organization criteria. Sociodemographic characteristics and oral health-related behaviours were collected using questionnaires. Multilevel zero-inflated negative binomial regression model was used to investigate the association between dental caries severity and dental fluorosis, sociodemographic characteristics, and oral health-related behaviours.

**Results:**

The final analyses included 12,350 and 27,818 children surveyed in 2005 and 2015, respectively. The standardized prevalence of dental caries increased from 27.05% (95% confidence interval [CI], 24.25-28.85) in 2005 to 37.92% (95% CI, 34.94-40.90) in 2015, and the respective standardized mean decayed, missing, filled teeth (DMFT) index scores increased from 0.50 (standard deviation [SD], 1.04) to 0.83 (SD, 1.45) (P < 0.001). Fujian province had the highest increase in dental caries, followed by Liaoning, Heilongjiang, Hainan, and Yunnan. Results revealed that children who were girls, more frequently experienced dental pain, and had more recent dental visits, had significantly higher DMFT scores after adjusting for the survey year and other variables (all P < 0.05).

**Conclusions:**

Dental caries of 12-year-old children in China deteriorated from 2005 to 2015, particularly in the northeast and southwest regions. Dental caries was associated with sex, dental pain, and dental service utilization.

**Supplementary Information:**

The online version contains supplementary material available at 10.1186/s12889-021-12262-x.

## Background

Dental caries, as one of the most common chronic diseases, remains a major public health burden worldwide [1]. According to the Global Burden of Disease Study 2016 (GBD 2016), dental caries of permanent teeth have the highest prevalence and the second highest incidence [2]. Oral health has not improved during the past three decades; the number of people with untreated oral conditions (including untreated dental caries, severe chronic periodontitis, and total tooth loss) dramatically increased from 2.5 billion in 1990 to 3.5 billion in 2015 [3].

Dental caries is a multifactorial and dynamic disease [4]. It shares common risk factors with other non-communicable diseases, including the consumption of free sugar, and their underlying social and commercial determinants of health [5, 6]. Dental caries impacts the quality of life; for example, they lead to reduced school days for children [5]. Twelve-year-old children have the early stage of permanent dentition, and their oral health status influences their following decades of life. Therefore, it is essential to prevent and detect oral diseases at an early stage.

Four national oral health surveys were conducted in China in 1983, 1995, 2005, and 2015. There are several books and articles describing the dental caries status of 12-year-old children based on data from a single national survey [7–13]. Two studies reported the trends of dental caries of school children based on the data of the GBD 2016 and those of the series of Chinese National Surveillance on Students’ Constitution and Health from 1991 to 2005 [14, 15]. No study has reported recent trends of dental caries in China based on the national survey data or explored risk indicators of dental caries on a national scale. Therefore, this study aimed to present the trends of dental caries among 12-year-old children in China from 2005 to 2015, and to explore their association with sociodemographic characteristics and oral health-related behaviours based on the latest two rounds of national surveys.

## Methods

### Data sources and procedure

This manuscript follows STROBE (Strengthening The Reporting of Observational Studies in Epidemiology) guidelines.

The present study used data obtained from the latest two rounds of the national oral health surveys conducted in China in 2005 and 2015. Both surveys were organized by the National Health Commission of the People’s Republic of China. For both surveys, 12-year-old participants were randomly selected from schools. The 2015 survey included all 31 provinces, autonomous regions, and municipalities in mainland China, whereas the 2005 survey included 30 of them. For both surveys, ethical approval was obtained from the Ethics Committee of the Chinese Stomatological Association. Written informed consent was obtained from the children’s parents or guardians prior to the implementation of data collection.

A multistage stratified sampling method was adopted for both surveys, the sampling scheme of which have been published elsewhere and was included in Appendix [9, 16]. Briefly, each province was regarded as a sampling unit in the first stage. Then, urban and rural districts were randomly selected from each province in the second stage. In the third stage, streets or towns were randomly selected from urban or rural districts. Finally, junior high schools were randomly selected from streets or towns. Afterwards, a certain number of students (equal boys and girls) were randomly selected from each school. The wholistic sampling methods used in 2005 and 2015 were similar, except for differences in the sample size of each sampling stage. In the 2005 and 2015 survey, a total of 21,600 and 29,760 children were invited respectively. Each survey consisted of two parts: a clinical oral examination and a questionnaire survey. All participants in the 2005 and 2015 surveys were clinically examined. In the 2005 survey, due to limitation of resources, only half of the participants were randomly selected to complete a questionnaire (selection bias not found after post-hoc analysis). In 2015, all participants were invited to complete a questionnaire. In this study, only records with both clinical examination and questionnaire data were used for the data analysis.

The criteria for the diagnosis of dental caries in the 2005 and 2015 surveys were the same. Dental caries experience of children were measured by the decayed, missing, filled teeth (DMFT) score by counting the number of teeth that were decayed, missing due to caries, and filled according to the recommendation of the WHO [17] . Those with DMFT score > 0 were considered as having dental caries experience, and the prevalence of dental caries was therefore calculated. The severity of dental caries was described by mean DMFT score. Dental fluorosis was recorded using the Dean’s index recommended by the WHO in both surveys [17]. Child with Dean’s index more than 0 was considered to have dental fluorosis. In both surveys, centralized and standardized training programmes were launched to ensure reliability of the results. All potential examiners from each province were calibrated against an experienced oral epidemiologist, and only those with kappa values higher than 0.8 for the assessment of dental caries experience were qualified to be an examiner for the survey. During the field survey, 5% of the participants were randomly selected for a duplicate examination to monitor inter-examiner reproducibility. Kappa values for the inter-examiner reliabilities were higher than 0.8 [16, 18].

After the clinical examination, children were instructed to self-complete a structured questionnaire. Not all questions in the questionnaires of the two rounds of surveys were identical. Only those questions sharing common wording and definitions across both surveys were selected for the data analysis of the present study. Information regarding sociodemographic characteristics (sex, ethnicity, location of residence, only child or not, parental highest educational attainment) and oral health-related behaviours (toothbrushing frequency, use of dental floss, frequency of sweet snack intake, time of the last dental visit, and frequency of toothache within the past 12 months) were obtained as indicator variables.

### Data analysis

The standardized prevalence of dental caries and mean DMFT score of the surveyed children in 2005 and those in 2015 were estimated using post-stratification weight (details are provided in Appendix). The standard error (SE) and standard deviation (SD) were estimated using the Taylor series linearization method. Due to skewed distribution of DMFT score, the Significant Caries (SiC) Index was calculated, which is the mean DMFT of the one third of the participants with the highest caries score [19]. The design-adjusted Rao-Scott *χ*^*2*^ test, *t*-test, and analysis of variance (ANOVA) were used to compare the prevalence and means, as appropriate.

Risk indicators of the individual level (level 1) and province level (level 2) in the 2005 and 2015 surveys were considered. Due to the nested structure of the data, multilevel models for multiple regression were performed; according to the likelihood ratio tests, they outperformed non-multilevel models. For the DMFT scores, due to overdispersion (variance exceeded the mean) and inflation of zero scores (71.30% of zeros in 2005 and 61.52% of zeros in 2015), the zero-inflated negative binomial model was preferred than negative binomial regression model or Poisson regression model according to the Vuong test. Thus, the multilevel zero-inflated negative binomial regression model was used to investigate the associations with individual-level and province-level variables. The zero-inflated model is comprised of two sub-models: the logistic regression model and the negative binomial regression model. A logistic regression model was used to predict sampling zeros and structural zeros, while the negative binomial model was used to predict count data. The logistic model and negative binomial model shared the same independent variables, and both included random intercepts. The individual-level variables were sex, ethnicity, residence location, only child or not, parental highest education attainment, toothbrushing frequency, use of dental floss, toothache within the past 12 months, time of the last dental visit, dental fluorosis, and frequency of sweet snack intake. The year of survey was introduced as a fixed effect to distinguish between the 2005 and 2015 survey participants. Province-level variables were GDP per capita quartiles and geographic regions. GDP per capita (per CNY 10,000), quartile 1 was defined as 0.51 to 0.94 for 2005, 2.62 to 3.67 for 2015, quartile 2 was defined as 0.99 to 1.13 for 2005, 3.68 to 4.13 for 2015, quartile 3 was defined as 1.14 to 1.86 for 2005, 4.28 to 6.54 for 2015, and quartile 4 was defined as 1.90 to 5.15 for 2005, 6.75 to 10.80 for 2015. Collinearity was ruled out by checking the variance inflation factor and correlation matrix. Estimated parameters were prevalence rate ratios (PRRs) and their corresponding 95% confidence intervals. No missing value imputation was performed. All statistical analyses were performed with SAS 9.4 and R 3.6.2, and all statistical testings were two-sided, at a significance level of 0.05.

## Results

A total of 12,392 children and 27,821 children who were 12 years old completed both the clinical examination and the questionnaire survey in 2005 and in 2015, respectively. After data cleaning, 12,350 records from the 2005 survey and 27,818 records from the 2015 survey were used in the final data analysis.

Most of the sociodemographic characteristic and oral health-related behaviour variables were statistically significantly different between the 2005 and 2015 survey samples (Table 1). Distributions of children according to sex, ethnicity, toothbrushing frequency, dental fluorosis, and province-level variables were not significantly different between the 2005 and 2015 survey participants. However, the proportions of children who were rural residents, had parents with higher education attainment, used dental floss, had more recent last dental visits, and took sweet snacks more frequently were higher in 2015 than those in 2005 (all P < 0.05). Moreover, the proportions of children who was the only child and had more frequent toothaches within the past 12 months were lower in 2015 (all P < 0.001).

**Table 1:** Distribution of socio-demographic characteristics and oral health-related behaviors in children aged 12 years in China in 2005 and 2015 surveys

	2005		2015		Total	
	(n = 12350)		(n= 27818)		(n = 40168)	
	Frequency	%	Frequency	%	Frequency	%
Level-1 (individual) indicators						
Sex						
Male	6330	51.26	13840	49.75	20170	50.21
Female	6020	48.74	13978	50.25	19998	49.79
Ethnicity						
Han	10963	88.77	24333	87.47	35296	87.87
Other	1387	11.23	3485	12.53	4872	12.13
Location of residence *						
Urban	5987	48.48	11522	41.42	17509	43.59
Rural	6363	51.52	16296	58.58	22659	56.41
Only child *						
Yes	5585	45.26	10009	35.99	15594	38.84
No	6755	54.74	17805	64.01	24560	61.16
Parental highest education attainment *						
Junior high school or below	1721	13.94	3306	11.88	5027	12.52
High school	7935	64.27	16421	59.03	24356	60.64
Tertiary or bachelor degree or above	2169	17.57	5335	19.18	7504	18.68
Unknown or orphan	521	4.22	2756	9.91	3277	8.16
Toothbrushing frequency						
Twice or more per day	3500	28.35	8879	31.93	12379	30.83
Once a day or less	8846	71.65	18933	68.07	27779	69.17
Use of dental floss *						
No or do not know	11436	92.73	25146	90.41	36582	91.12
Yes	897	7.27	2666	9.59	3563	8.88
Frequency of toothache within the past 12 months *						
Often	406	3.3	826	2.97	1232	3.07
Occasionally	5318	43.17	14518	52.19	19836	49.42
Never	4509	36.6	9226	33.17	13735	34.22
Can't remember	2086	16.93	3245	11.67	5331	13.28
Time of last dental visit *						
Within 6 months	1235	10.01	3645	13.1	4880	12.15
Within 6 to 12 months	1301	10.55	3704	13.32	5005	12.47
More than 12 months	3946	31.99	7432	26.72	11378	28.34
Never	5853	47.45	13035	46.86	18888	47.04
Dental fluorosis						
Yes	2264	18.37	5751	20.92	8015	20.13
No	10060	81.63	21741	79.08	31801	79.87
Frequency of sweet snack intake *						
Occasionally/ never	2853	23.1	7296	26.23	10149	25.27
Less than 2 times per week	4500	36.44	10946	39.35	15446	38.46
2 to 6 times per week	1924	15.58	5178	18.61	7102	17.68
More than 6 times per week	3071	24.87	4398	15.81	7469	18.6
Level-2 (province) indicators						
Economic development						
GDP per capita, quartile 1	2876	23.29	7270	26.13	10146	25.26
GDP per capita, quartile 2	2935	23.77	6093	21.9	9028	22.48
GDP per capita, quartile 3	3350	27.13	7093	25.5	10443	26
GDP per capita, quartile 4	3189	25.82	7362	26.46	10551	26.27
Region						
North China	1970	15.95	4473	16.08	6443	16.04
Northeast China	1184	9.59	2584	9.29	3768	9.38
East China	2916	23.61	6536	23.5	9452	23.53
Middlesouth (Central) China	2694	21.81	5188	18.65	7882	19.62
Southwest China	1549	12.54	4562	16.4	6111	15.21
Northwest China	2037	16.49	4475	16.09	6512	16.21

GDP = Gross domestic product (per CNY 10,000)

* p-values were less than 0.05 calculated using Rao-Scott *χ*^2^ test considering clustering

**Fig. 1:**
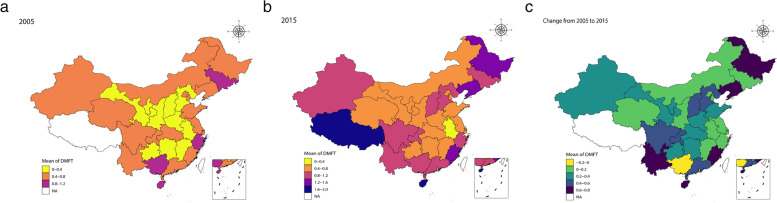
Dental caries levels (standardized DMFT scores) of children aged 12 years in China in 2005 and 2015 survey. **a** Dental caries levels (standardized DMFT scores) of children aged 12 years in China in 2005 survey. NA = Not Applicable. **b** Dental caries levels (standardized DMFT scores) of children aged 12 years in China in 2015 survey. NA = Not Applicable. **c** Change of dental caries levels (standardized DMFT scores) of children aged 12 years in China in 2005 and 2015 survey. NA = Not Applicable

Standardized dental caries outcomes of the surveyed children in 2005 and in 2015 are shown in Table 2, along with bivariate analysis results. The standardized prevalence of dental caries had increased from 27.05% (95% CI 24.25-28.85) in 2005 to 37.92% (95% CI 34.94-40.90) in 2015, and the respective standardized mean DMFT scores increased from 0.50 (SD = 1.04) to 0.83 (SD = 1.45) (P < 0.001). Untreated decayed teeth (DT) accounted for 89.38% and 83.38% of the dental caries experience of the children surveyed in 2005 and in 2015, respectively. The frequency distribution of the surveyed children according to the DMFT score was highly skewed with excessive zero scores in both surveys. The SiC Index increased from 1.61 to 2.42 over a decade. The national map with data regarding the severity of dental caries of 12-year-old children in 2005 and in 2015 is presented in Fig. 1a to 1c. In 2005 (Fig. 1a), the mean DMFT scores ranged from 0.26-1.10 among the 30 provinces, autonomous regions, and municipalities, and the overall national mean DMFT score was lower than 1.20. Guangxi (1.10), Hainan (1.07), and Jilin (1.06) provinces had the highest mean DMFT scores.

**Table 2:** Bivariate analysis of socio-demographic characteristics, oral health related behaviors with standardized prevalence of dental caries and mean DMFT scores in children aged 12 years in China in 2005 and 2015 surveys

	Prevalence of dental caries ^a^		DMFT scores ^b^	
	2005 (n=12350)	2015 (n=27818)	2005 (n=12350)	2015 (n=27818)
	Prevalence (%) and 95% CI	Prevalence (%) and 95% CI	Mean (SD)	Mean (SD)
Total	27.05 (24.25-28.85)	37.92 (34.94-40.90)	0.50 (1.04)	0.83 (1.45)
Level-1 (individual) indicators				
Sex				
Male	23.50 (20.58-26.42)***	33.40 (30.75-36.04)***	0.41 (0.95)***	0.67 (1.25)***
Female	30.83 (27.68-33.97)	42.67 (39.17-46.17)	0.58 (1.13)	1.00 (1.62)
Ethnicity				
Han	27.07 (24.32-29.81)	37.52 (34.52-40.52)***	0.49 (1.01)	0.82 (1.44)*
Other	26.89 (18.38-35.41)	44.12 (39.52-48.73)	0.57 (1.34)	1.01 (1.63)
Location of residence				
Urban	28.56 (26.02-31.11)	37.39 (33.29-41.49)	0.52 (1.02)	0.84 (1.47)
Rural	26.55 (23.44-29.67)	38.14 (35.13-41.15)	0.49 (1.05)	0.83 (1.44)
Only child				
Yes	27.91 (24.74-31.07)	36.99 (32.75-41.24)	0.51 (1.04)	0.82 (1.45)
No	26.55 (23.32-29.79)	38.35 (35.26-41.44)	0.49 (1.05)	0.84 (1.45)
Parental highest education attainment				
Junior high school or below	26.37 (22.27-30.47)**	36.73 (31.75-41.72)	0.48 (0.97)*	0.78 (1.39)
High school	26.37 (23.46-29.28)	38.39 (35.52-41.26)	0.48 (1.02)	0.84 (1.45)
Tertiary or bachelor degree or above	29.99 (27.05-32.92)	36.17 (33.20-39.14)	0.58 (1.21)	0.82 (1.49)
Unknown or orphan	32.94 (28.03-37.85)	38.87 (34.28-43.47)	0.62 (1.08)	0.87 (1.48)
Toothbrushing frequency				
Twice or more per day	29.13 (25.51-32.75)**	39.85 (36.75-42.95)**	0.53(1.02)	0.92(1.57)**
Once a day or less	26.44 (23.79-29.09)	37.08 (33.97-40.19)	0.49(1.05)	0.79(1.39)
Use of dental floss				
No or do not know	27.30 (24.57-30.04)	37.71 (34.82-40.60)	0.50 (1.05)	0.82 (1.44)*
Yes	23.76 (18.11-29.40)	40.29 (35.34-45.25)	0.43 (0.95)	0.96 (1.62)
Frequency of toothache within the past 12 months				
Often	39.19 (30.82-47.56)***	54.48 (49.27-59.68)***	0.77 (1.24)***	1.50 (1.97)***
Occasionally	30.68 (26.97-34.39)	42.55 (39.26-45.84)	0.59 (1.16)	0.98 (1.58)
Never	22.45 (19.89-25.02)	30.29 (27.30-33.28)	0.38 (0.86)	0.58 (1.15)
Can't remember	25.04 (21.42-28.67)	34.34 (30.58-38.11)	0.45 (1.01)	0.71 (1.32)
Time of last dental visit				
Within 6 months	32.13 (26.57-37.69)***	48.76 (43.95-53.58)***	0.63 (1.19)***	1.27 (1.89)***
Within 6 to 12 months	31.79 (27.38-36.21)	46.20 (43.44-48.96)	0.59 (1.21)	1.07 (1.59)
More than 12 months	30.52 (27.28-33.76)	40.24 (36.72-43.76)	0.58 (1.12)	0.88 (1.48)
Never	23.74 (21.29-26.19)	31.67 (28.87-34.48)	0.42 (0.94)	0.63 (1.21)
Dental fluorosis				
Yes	24.20 (20.98-27.43)*	37.10 (33.89-40.31)	0.41 (0.96)**	0.76 (1.33)
No	27.71 (24.75-30.68)	38.04 (34.72-41.35)	0.52 (1.06)	0.84 (1.48)
Frequency of sweet snack intake				
Occasionally/ never	25.14 (22.50-27.78)***	34.00 (30.68-37.33)***	0.48 (1.05)**	0.69 (1.25)***
Less than 2 times per week	25.01 (22.64-27.38)	36.21 (33.10-39.33)	0.44 (0.99)	0.77 (1.38)
2 to 6 times per week	29.79 (26.23-33.35)	38.61 (35.68-41.53)	0.54 (1.01)	0.85 (1.49)
More than 6 times per week	29.07 (25.25-32.90)	40.40 (36.90-43.91)	0.54 (1.08)	0.93 (1.55)
Level-2 (province) indicators				
Economic development				
GDP per capita, quartile 1	24.48 (17.11-31.86)**	34.84 (24.70-44.98)	0.48 (1.14)*	0.74 (1.33)*
GDP per capita, quartile 2	21.53 (18.51-24.55)	40.25 (32.32-48.19)	0.36 (0.82)	0.89 (1.51)
GDP per capita, quartile 3	29.56 (24.01-35.11)	38.07 (32.53-43.62)	0.55 (1.09)	0.82 (1.40)
GDP per capita, quartile 4	30.82 (25.90-35.74)	38.20 (32.68-43.71)	0.56 (1.03)	0.86 (1.54)
Region				
North China	25.46 (22.75-28.17)*	36.42 (29.81-43.03)***	0.41 (0.86)	0.76 (1.32)***
Northeast China	36.12 (22.46-49.78)	51.33 (40.43-62.24)	0.73 (1.31)	1.31 (1.86)
East China	28.37 (21.46-35.28)	34.45 (26.83-42.06)	0.50 (0.99)	0.74 (1.39)
Middlesouth (Central) China	27.23 (18.41-36.06)	38.39 (32.69-44.09)	0.52 (1.10)	0.82 (1.42)
Southwest China	22.50 (17.30-27.70)	40.83 (33.82-47.83)	0.43 (1.06)	0.93 (1.53)
Northwest China	22.70 (13.72-31.68)	31.68 (20.29-43.07)	0.38 (0.86)	0.63 (1.22)

CI = Confidence interval; SD = Standard deviation; GDP = Gross domestic product (per CNY 10,000);

DMFT = Decayed, Missing, and Filled Teeth

^a^Values were calculated comparing prevalence between years using Rao-Scott *χ*^2^ test considering weight and clustering

^b^p-values were calculated comparing means of DMFT scores between years using *t*-test or ANOVA considering weight and clustering

* P < 0.05; ** P <0.01; *** P < 0.001

In 2015 (Fig. 1b), the mean DMFT scores ranged from 0.35-1.83 among the 31 provinces, autonomous regions, and municipalities. Among them, five had a mean DMFT score greater than 1.20, whereas Tibet (1.83), Hainan (1.80), and Heilongjiang (1.45) had the highest mean DMFT scores. As shown in Fig. 1c, a reduction in the severity of dental caries between 2005 and 2015 was observed only in Guangxi province; the dental caries status in the other 30 provinces, autonomous regions, and municipalities had worsened. The provinces with the greatest increases in mean DMFT scores were Fujian (0.79), Liaoning (0.79), Heilongjiang (0.76), Hainan (0.72), and Yunnan (0.66).

**Fig. 2:**
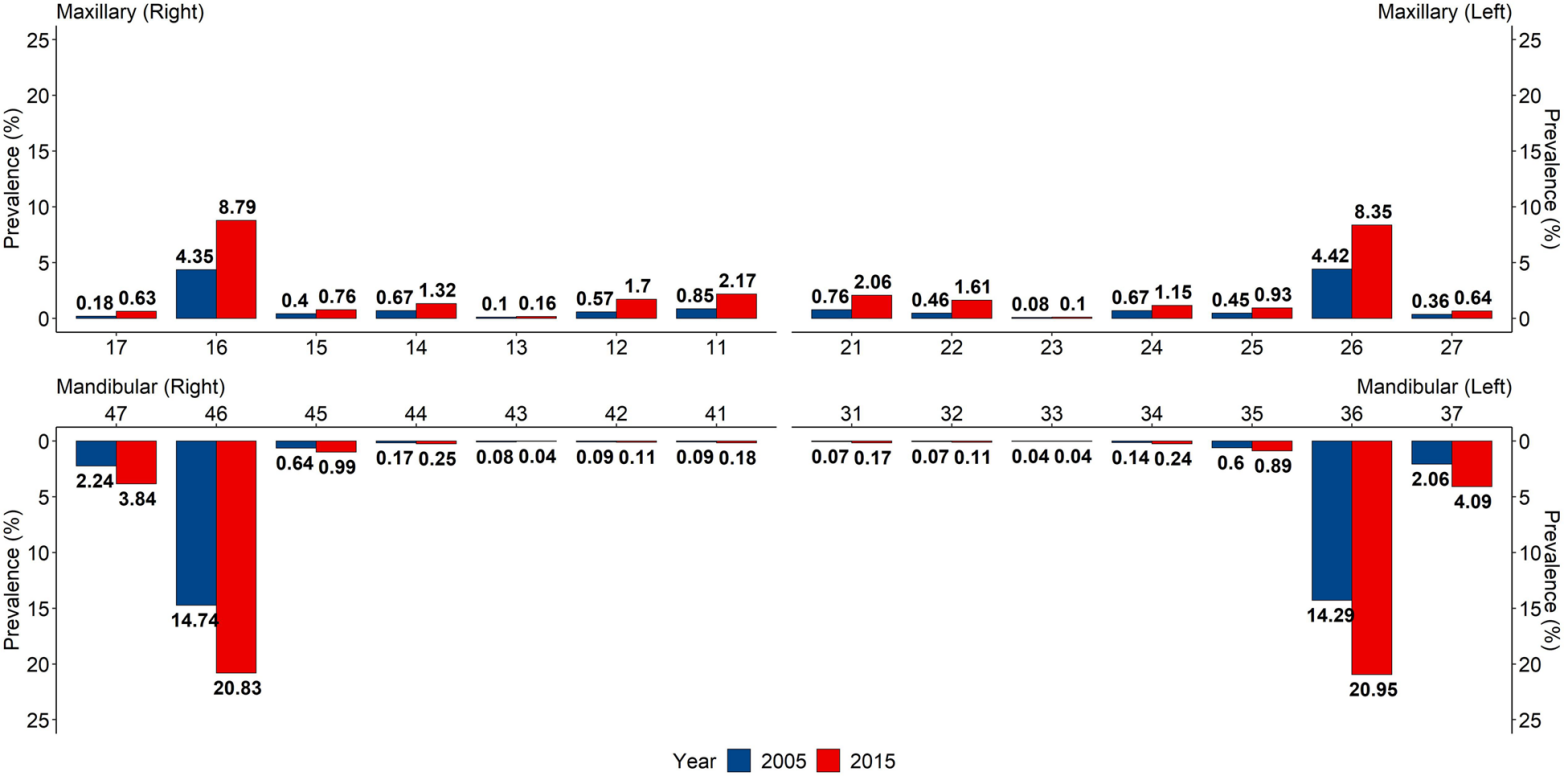
Comparison of prevalence of dental caries of each tooth of children aged 12 years old in China in 2005 and 2015 survey

The four first permanent molar teeth had the highest prevalence of dental caries in both the 2005 and 2015 surveys, whereas the mandibular incisors had the lowest (Fig. 2). Among the anterior teeth, the prevalence in maxillary teeth was higher than that in mandibular teeth, whereas the situation was the opposite among the posterior teeth. The prevalence of dental caries for the mandibular first molar increased over 6% from 2005 to 2015, followed by the maxillary first molar (increased around 4%), mandibular second molar (increased over 1.6%) and maxillary first incisors (increased by over 1.3%).

In all the multilevel models of the DMFT score (Table 3), larger DMFT scores were found among children who were girls, had more frequent toothaches within the past 12 months, and had more recent last dental visits (all P < 0.0001). In the multilevel model of 2005 data, no province-level factor was significantly associated with the DMFT score. In the multilevel model of 2015 data, children who were of non-Han ethnicity and took sweet snacks more frequently had significantly higher DMFT scores (all P < 0.05). Moreover, one of the province-level factors (regions) was significantly associated with DMFT scores, with children from northeast and southwest China having higher DMFT scores than those of children from east China (P < 0.05). In the multilevel models of the combined 2005 and 2015 data, the significant variables were almost the same as those of the 2015 data model. DMFT scores of the children who participated in the 2015 survey were 1.30 times compared with those of the children who participated in the 2005 survey (P < 0.0001).

**Table 3:** Multilevel zero-inflated negative binomial regression models for DMFT score in children aged 12 years in China in 2005 and 2015 surveys

	2005 data		2015 data		2005 and 2015 combined	
	PRR (95% CI)	p value	PRR (95% CI)	p value	PRR (95% CI)	p value
**Level-1 indicators**						
Sex						
Female (*vs.* Male)	1.19(1.09-1.30)	0.0001	1.23(1.17-1.29)	<0.0001	1.22(1.17-1.27)	<0.0001
Ethnicity						
Other (*vs.* Han)	1.11(0.96-1.28)	0.15	0.96(0.88-1.04)	0.30	0.99(0.92-1.07)	0.78
Location of residence						
Rural (*vs.* Urban)	1.06(0.96-1.16)	0.24	1.01(0.96-1.07)	0.59	1.03(0.99-1.08)	0.14
Only child						
Yes (*vs.* No)	0.95(0.86-1.05)	0.29	0.96(0.92-1.01)	0.11	0.98(0.93-1.02)	0.33
Parental highest education attainment (*vs.* Junior high school or below)						
High school	1.05(0.92-1.19)	0.47	1.02(0.95-1.11)	0.54	1.04(0.97-1.11)	0.29
Tertiary or bachelor degree or above	1.11(0.94-1.30)	0.22	1.02(0.93-1.12)	0.63	1.04(0.96-1.13)	0.30
Unknown or orphan	1.06(0.85-1.31)	0.62	1.04(0.95-1.15)	0.39	1.05(0.96-1.15)	0.27
Toothbrushing frequency						
Twice or more per day (*vs.* Once a day or less)	0.92(0.83-1.01)	0.087	1.02(0.98-1.06)	0.40	1.01(0.97-1.06)	0.60
Use of dental floss						
Yes (*vs.* No or do not know)	0.95(0.80-1.12)	0.53	1.06(0.99-1.14)	0.098	1.05(0.98-1.12)	0.15
Frequency of toothache within the past 12 months (*vs.* Never)						
Often	1.40(1.15-1.70)	0.0008	1.45(1.30-1.62)	<0.0001	1.44(1.31-1.59)	<0.0001
Occasionally	1.25(1.12-1.39)	<0.0001	1.28(1.21-1.36)	<0.0001	1.27(1.21-1.33)	<0.0001
Can't remember	1.09(0.95-1.25)	0.21	1.08(1.00-1.18)	0.063	1.08(1.00-1.16)	0.037
Time of last dental visit (*vs.* Never)						
Within 6 months	1.23(1.07-1.43)	0.0047	1.43(1.33-1.52)	<0.0001	1.40(1.32-1.49)	<0.0001
Within 6 to 12 months	1.13(0.98-1.31)	0.095	1.25(1.17-1.34)	<0.0001	1.23(1.16-1.31)	<0.0001
More than 12 months	1.32(1.20-1.46)	<0.0001	1.17(1.10-1.24)	<0.0001	1.21(1.15-1.27)	<0.0001
Dental fluorosis						
Yes (*vs.* No)	1.08(0.95-1.22)	0.25	0.95(0.89-1.01)	0.11	0.98(0.92-1.03)	0.37
Frequency of sweet snack intake (*vs.* Occasionally/ never)						
Less than 2 times per week	0.92(0.81-1.05)	0.23	1.04(0.97-1.11)	0.29	1.00(0.93-1.07)	0.91
2 to 6 times per week	0.96(0.83-1.11)	0.58	1.09(1.02-1.17)	0.0073	1.03(0.96-1.10)	0.41
More than 6 times per week	0.93(0.82-1.04)	0.21	1.13(1.06-1.21)	<0.0001	1.06(0.99-1.13)	0.10
Year of survey						
2015 (*vs.* 2005)	..	..	..	..	1.30(1.23-1.37)	<0.0001
**Level-2 indicators**						
Economic development (*vs.* GDP per capita, quartile 1)						
GDP per capita, , quartile 2	0.98(0.75-1.29)	0.90	1.08(0.86-1.35)	0.53	1.01(0.90-1.14)	0.85
GDP per capita, , quartile 3	1.21(0.90-1.62)	0.21	0.96(0.76-1.20)	0.71	0.96(0.86-1.08)	0.52
GDP per capita, , quartile 4	1.13(0.85-1.51)	0.40	1.10(0.87-1.38)	0.44	0.88(0.77-1.01)	0.068
Region (*vs.* East China)						
North China	0.86(0.65-1.13)	0.27	0.93(0.73-1.19)	0.57	0.97(0.77-1.22)	0.80
Northeast China	1.15(0.84-1.57)	0.39	1.50(1.09-2.07)	0.014	1.35(1.03-1.77)	0.031
Middlesouth (Central) China	1.13(0.86-1.50)	0.38	1.18(0.92-1.52)	0.18	1.11(0.89-1.38)	0.37
Southwest China	1.27(0.91-1.78)	0.16	1.40(1.07-1.83)	0.013	1.26(0.99-1.59)	0.06
Northwest China	0.94(0.68-1.29)	0.69	1.05(0.79-1.39)	0.73	0.94(0.74-1.20)	0.64

PRR = Prevalence rate ratio

CI = Confidence interval

## Discussion

The present study describes the trend in dental caries status among 12-year-old children in China from 2005 to 2015 using national survey data. Our findings revealed that dental caries situation among the children aged 12 years had worsened from 2005 to 2015, particularly in the northeast and southwest regions.

Dental caries level of the 12-year-old age group is recommended by the WHO as a reference to assess the dental caries status of school-age children in different countries or regions [20]. The dental caries status among this age group in China is similar to those in many major developed countries while being lower than those in most of developing countries. The mean DMFT scores among 12-year-old children in China were 0.50 and 0.83 in 2005 and 2015. For comparison, according to the oral health country/area profile project database of the WHO, the mean DMFT scores of 12-year-old children were 1.2 in the United States, 1.4 in Japan, 1.0 in Canada, 0.8 in the United Kingdom, and 0.8 in Sweden [21]. The corresponding figures in developing countries were 2.5 in Russia, 2.1 in Brazil, 1.9 in Chile, 1.6 in India, and 1.3 in Thailand. According to the WHO criteria, the dental caries level in China is considered low (mean DMFT score < 1.2). The traditional Chinese diet is healthy and the relatively high intake of seafood in the southeast coast areas, which are rich in fluoride, may be beneficial to the prevention of dental caries [22]. However, both the nutrition status of people in China and their dietary habits, especially in rural areas, have changed recently and the diet has become more protein and sugar rich [23]. A sugar-rich diet and frequent intake of sugary snacks promote the development of dental caries, especially in children. More health education efforts are needed to combat these unhealthy dietary habits.

The multilevel statistical models adopted in the data analysis in the present study consistently showed that sex, toothache within the past 12 months, and the recency of the last dental visit were associated with the dental caries status of Chinese children surveyed in 2005 and 2015, even after adjusting for the potential confounding effects of other variables. Although there is no consensus in the dental literature that girls have a higher dental caries level than boys, several cohort and cross-sectional studies found a difference in sex [24, 25]. Suggested reasons for this include earlier tooth eruption in girls, more access to snacks, more frequent utilisation of dental services, and genetic susceptibility [26–28]. More oral health education and promotion should be provided to school children, especially female ones, in China.

It is not surprising that a higher level of dental caries was found among the surveyed Chinese children who had more frequent toothaches and more recent last dental visits [29, 30]. This finding is consistent with those of several studies on the risk indicators of dental caries [31, 32]. The possible reason is that these children visited the dentist only when they had a dental problem, such as a toothache, which was most probably due to advanced dental caries. The dental visit patterns of the people in China are mostly problem-oriented; they are less likely to attend regular dental check-up and preventive care. This may directly affect the economic burden of oral diseases in China [33]. A study on the global economic impact of dental diseases in 2015 showed that the direct cost of oral diseases in China (US $60.11 billion) ranked the second highest in the world, just after the United States (US $119.07 billion) [34]. Because the cost of preventive measures such as fissure sealants are much cheaper than curative treatment, the promotion of these measures among the general public is essential, along with proper amendments of medical insurance accounts related policies and regulations of preventive dental care services.

There are limitations. Firstly, only data from the two most recent national surveys were included in this study, an analysis of longer-term trends of dental caries of Chinese children was not performed. Secondly, the question regarding why dental caries increased has not been fully answered since cross-sectional studies are limited for causal inference. Thirdly, although around 7% people of all ages have never been to school according to the census report in 2010 in China, the present survey was conducted among school students, and selection bias may be existed because those adolescents who were not educated in schools were excluded [35].

Findings of this study have implications for practice. The 2015 oral health survey was the first national survey conducted in Tibet, which located in southwest China, and it is found that the dental caries status there was the worst among the 31 provinces, autonomous regions, and municipalities. A high increase in dental caries between 2005 and 2015 was found among children in Yunnan province, which is also located in the southwest region. It is noted that in these two places, there is a high proportion of non-Han ethnic groups in the population (91.83% in Tibet and 33.37% in Yunnan) [36, 37]. Meanwhile, Heilongjiang, Jilin, and Liaoning provinces, which are located in northeast China, also had a high dental caries level. More intensive oral health education and dental caries prevention programmes are probably needed in those places, especially for the non-Han ethnic groups.

It was also found that first permanent molar teeth had the highest prevalence of dental caries among all teeth in the surveyed children in both 2005 and 2015. This is most likely because these teeth have deep fissures in their occlusal surfaces and erupt in the mouth first. It is known that placement of fissure sealant is the most effective intervention for preventing caries in molar teeth [38]. The Chinese government has implemented the National Oral Health Comprehensive Intervention Program for children since 2008 to reduce dental caries [39]. The programme started in 22 provinces located in the central and western regions and comprises oral health education for children, regular dental check-ups, the provision of pit and fissure sealants for the first permanent molars of children aged 7-9 years, and training for the dental workforce. Results of the 2015 oral health survey show a significant difference in the dental caries status and oral health behaviours of the 12-year-old children in the programme-covered and uncovered regions [40]. The survey findings confirmed that a preventive approach for the entire population of children is more beneficial than a restorative approach [6]. However, a large proportion of school-age children in China are not yet covered by the aforementioned intervention programmes. Oral health care has always been an integral part of the healthcare system in China [41]. Health insurance accounts only cover basic dental treatments such as fillings and extractions while prevention measures such as fissure sealant and the application of topical fluoride varnish are not covered [42]. To achieve the goal that the prevalence of dental caries among 12-year-old children reducing to 30% by 2030, it is essential to invest in more resources to expand the coverage of the school-based oral health intervention programme and to allow the use of medical insurance accounts for dental preventive car e[43].. It is very likely that these proposed measures would reduce the inequality in the dental caries prevalence among Chinese school children and particularly benefit those living in the southwest and northeast regions [44].

## Conclusions

In conclusion, in China, dental caries level among 12-year-old children worsened in 2005 to 2015, particularly for those living in northeast and southwest China. The risk indicators for dental caries in Chinese school children are sex, toothache frequency, and recency of the last dental visit.

## Supplementary Information

**Additional file 1.**

## Data Availability

The data that support the findings of this study are available from Chinese Stomatological Association but restrictions apply to the availability of these data, which were used under license for the current study, and so are not publicly available. Data are however available from the authors upon reasonable request and with permission of Chinese Stomatological Association.
