# Supplementary material for: Trend on dental caries status and its risk indicators in children aged 12 years in China: a multilevel analysis based on the repeated national cross-sectional surveys in 2005 and 2015
Source: BMC Public Health. 2021 Dec 15;21:2285. doi: 10.1186/s12889-021-12262-x (PMC8672523; doi:10.1186/s12889-021-12262-x)

**Appendix**

**Title: Trend on dental caries status and its risk indicators in children aged 12 years in China: a multilevel analysis based on the repeated national cross-sectional surveys in 2005 and 2015**

**Authors**

Fei Li PhD *^a^, Si-Cheng Wu MPH *^b^, Zhi-Yuan Zhang MD ^c^, Edward Chin Man Lo PhD ^d^, Wen-Jia Gu ^a^, Dan-Ying Tao MD ^a^, Xing Wang MD ^e^, Bao-Jun Tai MD ^f^, De-Yu Hu MD ^g^, Huan-Cai Lin PhD ^h^, Bo Wang MD ^e^, Yan Si MD ^j^, Chun-Xiao Wang MD ^i^, Shu-Guo Zheng MD ^j^, Xue-Nan Liu MD ^j^, Wen-Sheng Rong PhD ^j^, Wei-Jian Wang MD ^j^, Xi-Ping Feng MD ^#a^, Hai-Xia Lu PhD ^#a^

^a^ Department of Preventive Dentistry, Shanghai Ninth People’s Hospital, Shanghai Jiao Tong University School of Medicine; College of Stomatology, Shanghai Jiao Tong University; National Center for Stomatology; National Clinical Research Center for Oral Diseases; Shanghai Key Laboratory of Stomatology, Shanghai, China

^b^ Biostatistics Office of Clinical Research Center, Shanghai Ninth People's Hospital, Shanghai JiaoTong University, School of Medicine; Shanghai, China

^c^ National Clinical Research Center for Oral Diseases, Shanghai Ninth People's Hospital, College of Stomatology, Shanghai JiaoTong University, School of Medicine; Shanghai, China

^d^ Dental Public Health, Faculty of Dentistry, University of Hong Kong, Hong Kong

^e^ Chinese Stomatological Association, Beijing, China

^f^ School & Hospital of Stomatology, Wuhan University, Wuhan, China

^g^ West China School of Stomatology, Sichuan University, Chengdu, China

^h^ Guanghua School of Stomatology, Hospital of Stomatology, Sun Yat-sen University, Guangzhou, China

^i^ Chinese Center for Disease Control and Prevention, Beijing, China

^j^ Department of Preventive Dentistry, Peking University School and Hospital of Stomatology, National Engineering Laboratory for Digital and Material Technology of Stomatology, Beijing Key Laboratory of Digital Stomatology, Beijing, China

* These authors contributed equally to this study and share first authorship

^#^ These authors contributed equally to this study and share corresponding authorship

1. **Detailed sampling methods for both surveys.**

A multistage stratified sampling method was adopted for both surveys. (Refer to Appendix Figure 1.)

In 2005, in the first stage, each province was regarded as a sampling unit. In the second stage, each province was divided into two strata: urban and rural districts. Urban districts were divided into three strata according to their size: large, medium, and small cities. Rural districts were divided into three strata according to their gross domestic product (GDP). Then, one city or county was randomly selected from each stratum. A total of 180 cities or counties were selected. In the third stage, three streets or townships (referred to as streets in urban districts and as townships in rural districts) were randomly selected (sorted by population size) in each sampled city or county. Thus, 540 streets or townships were selected. Finally, two junior high schools were randomly selected from each selected survey site; 20 students who were 12 years old (equal numbers of boys and girls) were randomly selected from each school. A total of 21,600 children from 1,080 schools were invited to participate in the survey.

In the 2015 survey, each province was regarded as a sampling unit in the first stage. In the second stage, two urban and two rural districts (defined according to the National Bureau of Statistics of China) were selected in each province by the local Centre for Disease Control and Prevention. Totally, 62 urban and 62 rural districts were selected. In the third stage, three streets in an urban district or three townships in a rural district were randomly selected from each district. A total of 186 streets and 186 townships were selected. Finally, one junior high school of each street or township was selected; 80 students who were 12 years old (equal numbers of boys and girls) from each school were randomly selected and invited to participate in the survey. A total of 29,760 children from 372 schools were invited.


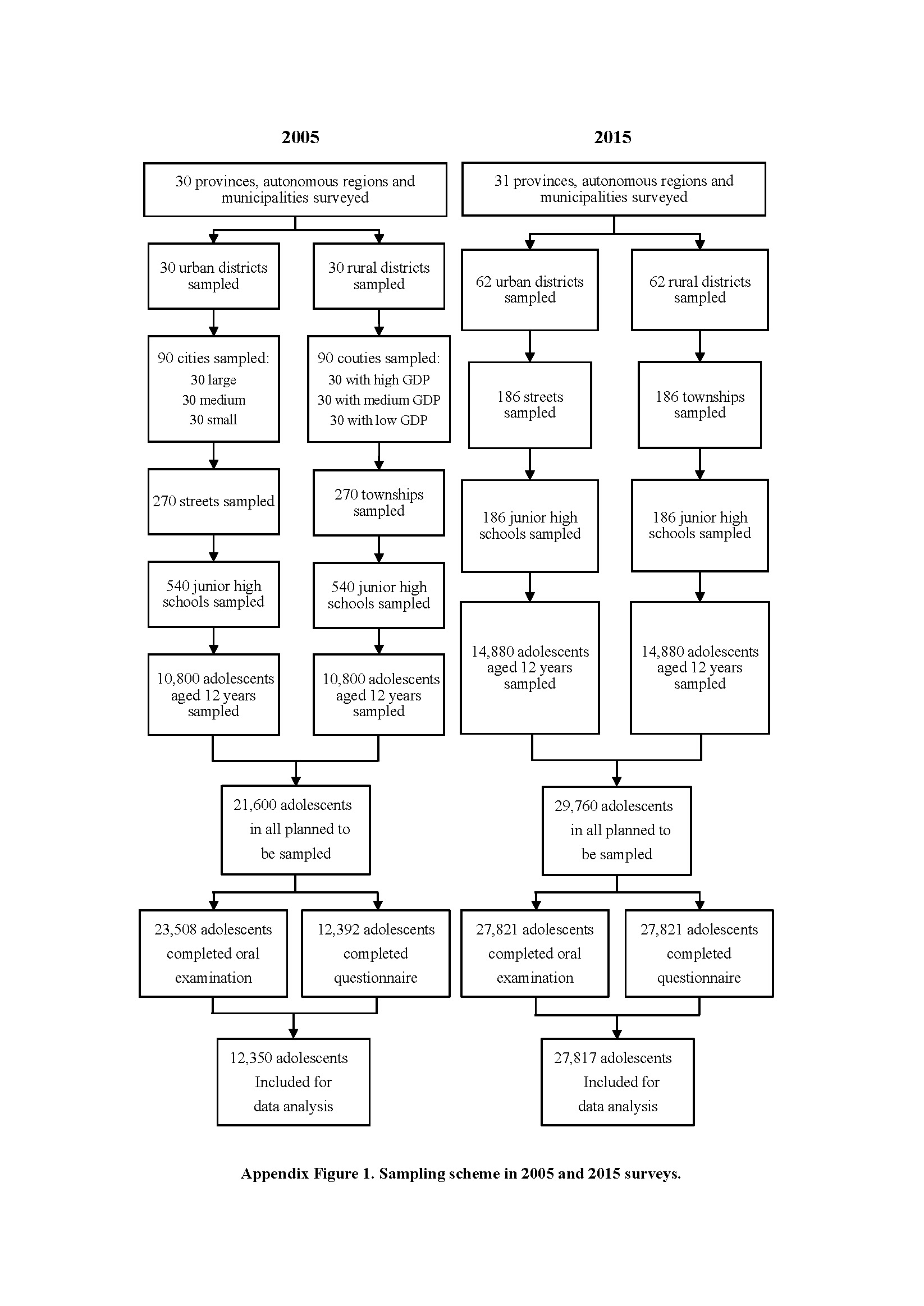


1. **Detailed method for post-stratification weight.**

The standardized prevalence of dental caries experience and mean DMFT score of the surveyed children in 2005 and those in 2015 were estimated. Post-stratification weight was used, which harmonized the sample structure of the survey with the standard population, particularly for province, sex, and residence. If any weighting variable (province, sex, and residence) was missing, then the records were removed from the final data analysis. Because standard population data of 12-year-old children in 2005 and in 2015 were not available, the standard population from the 2000 census and 2010 census of the Chinese population were used as alternatives.

1. **Distribution of DMFT Index of 12 years old adolescents in China (2005 and 2015).**


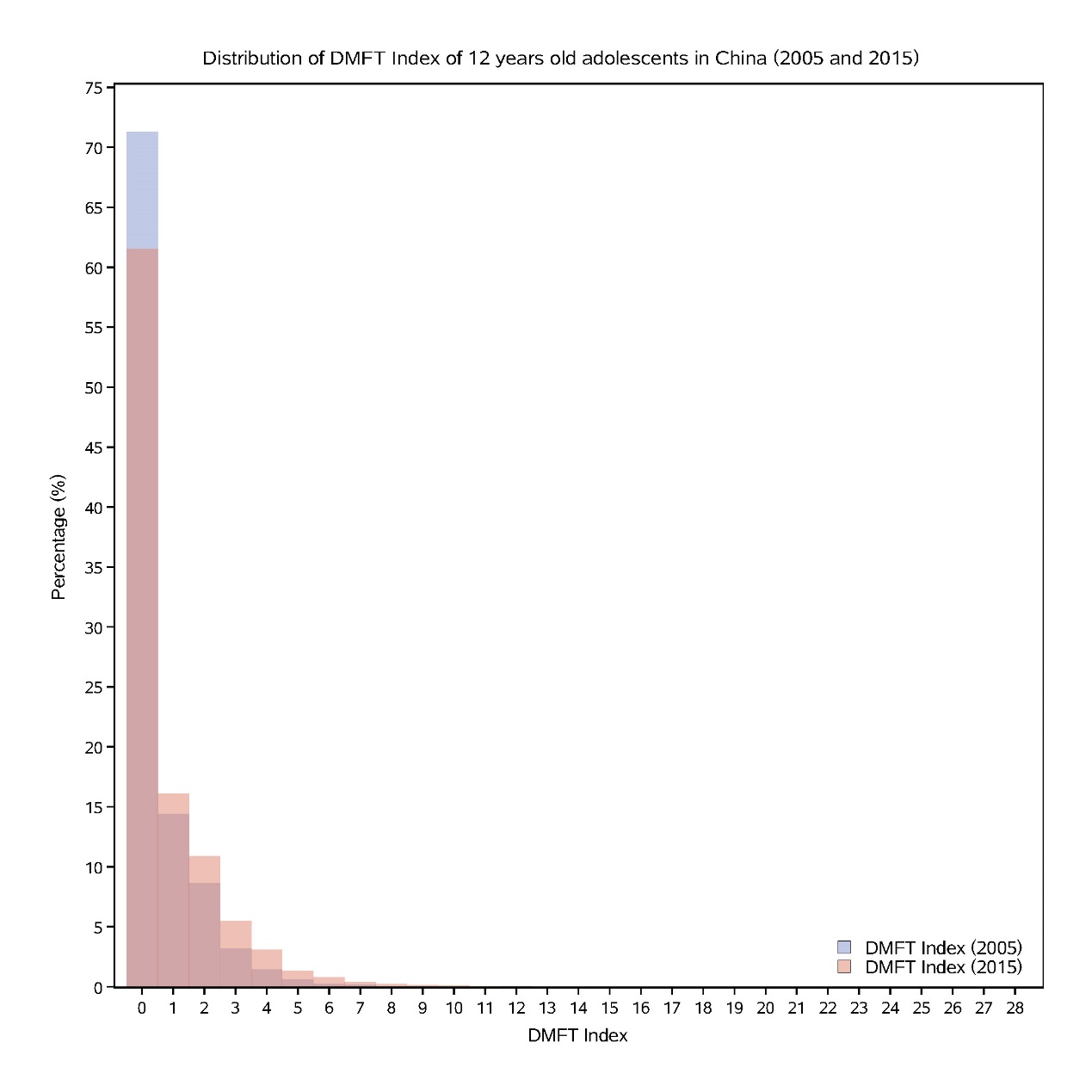


1. **Prevalence of “Decayed”, “Missing”, and “Filled” teeth of each tooth of children aged 12 years old in China in 2005 survey.**


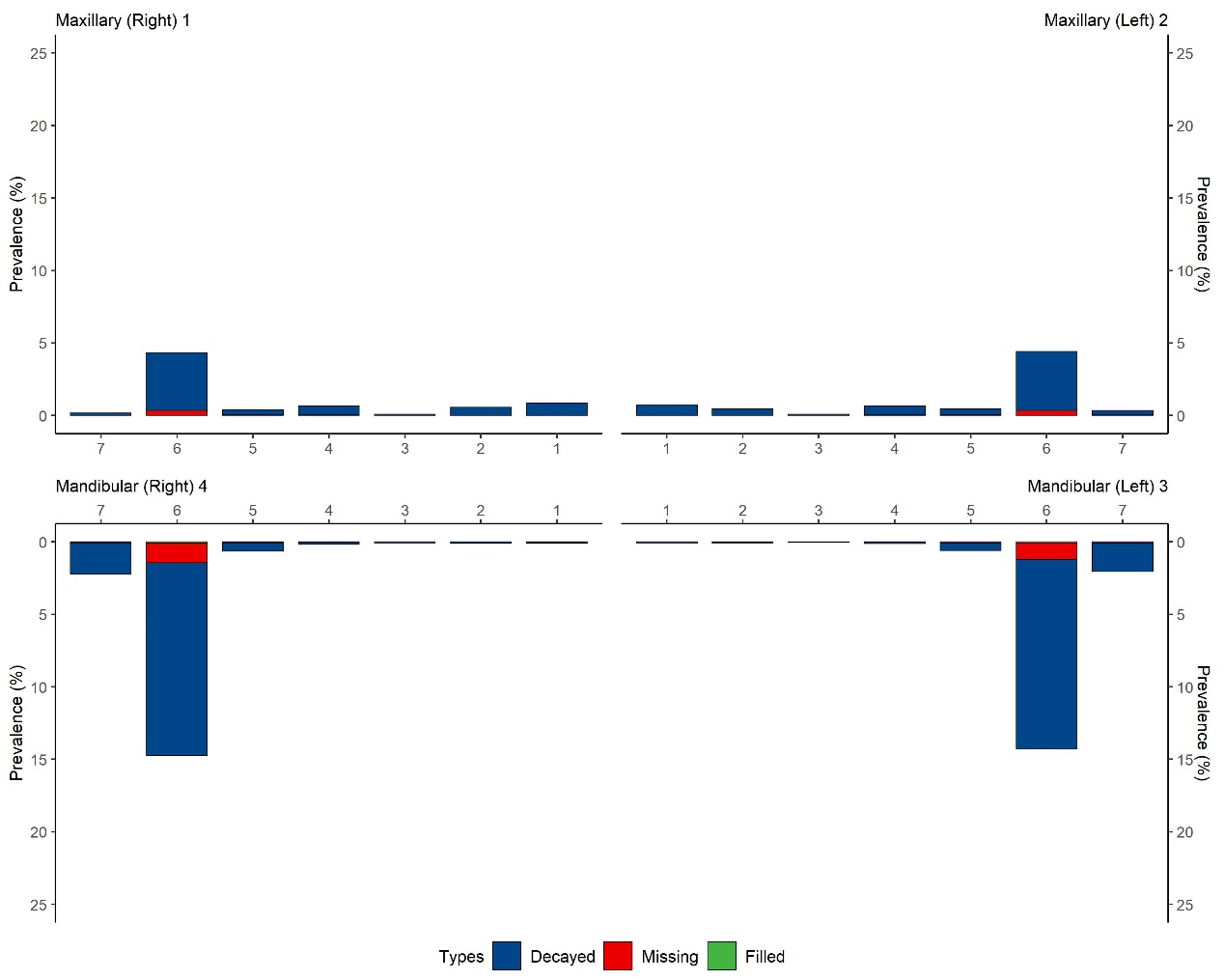


1. **Prevalence of “Decayed”, “Missing”, and “Filled” teeth of each tooth of children aged 12 years old in China in 2015 survey.**


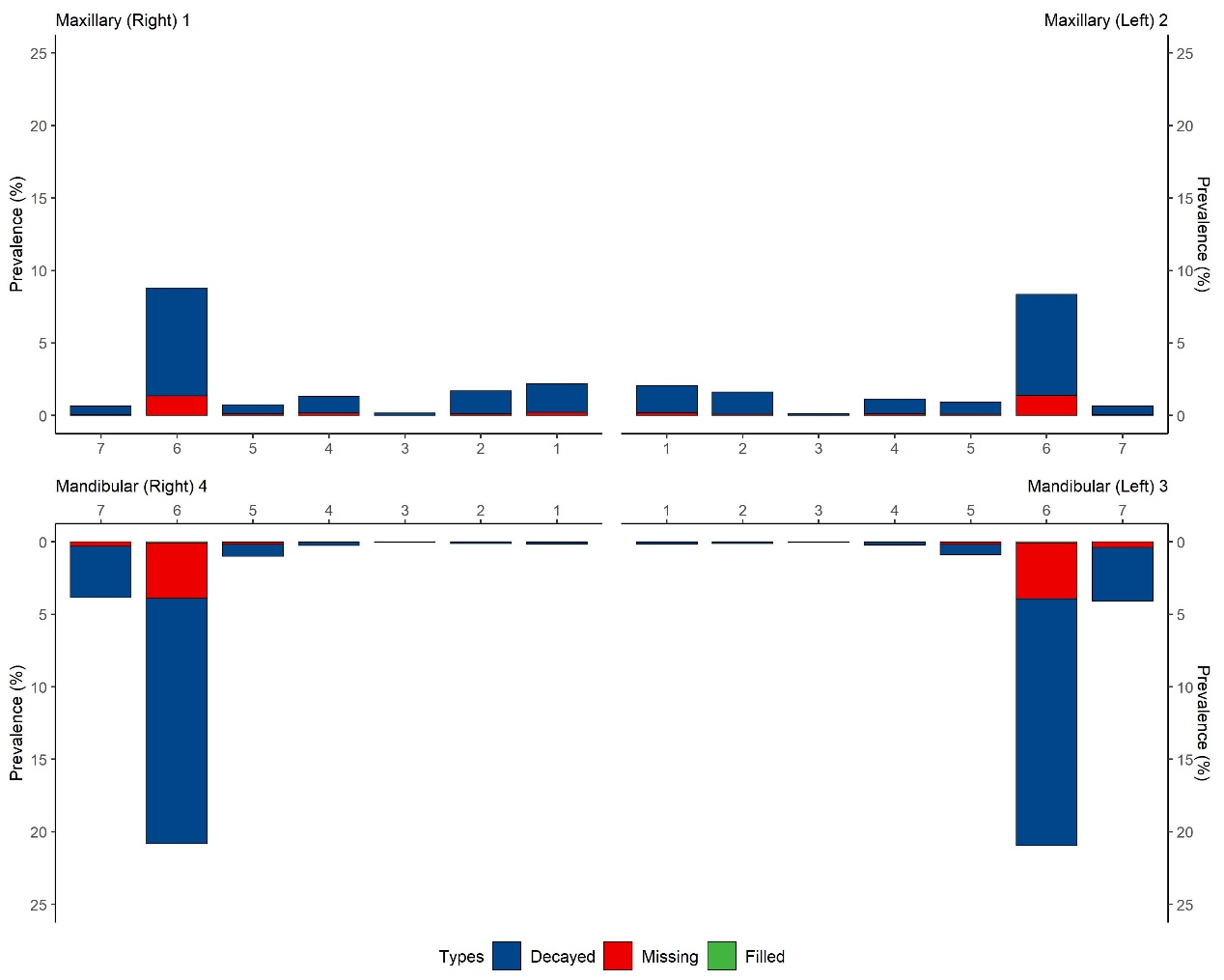

Supplement: Supplementary file 1 — Additional file 1. [file 12889_2021_12262_MOESM1_ESM.docx]
